# Adjustment of Matrix Effects in Analysis of 36 Secondary Metabolites of Microbial and Plant Origin in Indoor Floor Dust Using Liquid Chromatography-Tandem Mass Spectrometry

**DOI:** 10.3390/buildings13051112

**Published:** 2023

**Authors:** Cornelius Rimayi, Ju-Hyeong Park

**Affiliations:** Respiratory Health Division, National Institute for Occupational Safety and Health, Centers for Disease Control and Prevention, Morgantown, WV 26505, USA

**Keywords:** indoor floor dust, matrix effect adjustment, microbial secondary metabolite, recovery

## Abstract

Exposure to microbial agents in water-damaged buildings is a major public health concern. Liquid chromatography-tandem mass spectrometry (LC-MS/MS) has become a primary tool for testing environmental samples for microbial secondary metabolites (SMs); however, matrix effects can lead to inaccurate results in exposure assessment. Applying a universal internal standard (ISTD) and a matrix-matched calibration can adjust for matrix effects, as shown by our previous study. However, there are only few isotope-labeled internal standards for SMs available on the market. In this study, we determined the best-performing ISTDs among ten candidates (nine ^13^C-labeled isotopes and one unlabeled analogue) for each of 36 SMs. We analyzed school floor dust spiked with the 36 SMs to identify the best-performing ISTDs (initial experiment) and examined reproducibility with the selected ISTDs and the same spiked dust (validation 1). We also tested applicability for the selected ISTDs using spiked dust collected from different schools (validation 2). The three experiments showed that 26, 17, and 19 SMs had recoveries within the range 100 ± 40%. ^13^C-ochratoxin A and ^13^C-citrinin were most frequently selected as the best ISTDs for the 36 SMs, followed by deepoxy-deoxynivalenol, ^13^C-sterigmatocystin, and ^13^C-deoxynivalenol. Our study shows that using the identified, best-performing analogous ISTDs for those metabolites may improve testing accuracy for indoor dust and help better estimate exposure effects on potential health.

## 1. Introduction

Building materials containing hydrocarbons can promote microbial growth in damp or water-damaged indoor environments resulting from improper building maintenance, poor ventilation, defective building design, or natural disasters such as flooding from hurricanes [1,2]. Exposure to dampness and mold in such indoor environments has been associated with various respiratory illnesses [3–6] and has become a major public health concern over the last 40 years [4,5].

Although the causal microbial agents for specific health effects are currently not well understood [3], recent studies examining associations of health effects with exposure to microbial or other secondary metabolites (SMs) have received researchers’ attention [7,8]. In exposure assessments for such epidemiological studies, accurate measurement of SMs in samples from damp or moldy buildings is crucial for a correct understanding of the role of SMs in potential health outcomes.

In the analysis of SMs in environmental samples, liquid chromatography-tandem mass spectrometry (LC-MS/MS) has become a primary tool due to its high sensitivity when quantifying small molecules at very low concentrations [6,9]. However, as noted in our previous study [10], one of the limitations of the method is substantial matrix effects in which the ionization efficiency of the target analytes is negatively influenced by coeluting substances from the sample matrix.

Researchers may adjust these matrix effects using one of three methods: (1) preparing a standard curve in the sample matrix that is being tested (matrix matching); (2) adding standard materials to the sample (standard addition); or (3) using internal standards (ISTDs) to compensate for the loss from extraction and inefficient ionization [11]. Each method has its drawbacks. Matrix-matched calibration must be specific to the individual sample matrix, which changes from sample to sample [12]. In addition, sample media used in matrix-matched calibration should not contain the analytes to be quantified, which makes the application of this method difficult. Adding standard materials to each sample using the standard addition method compensates for sample-specific matrix effects but increases the sample size at least twofold [13]. The ISTD adjustment method uses stable, isotope-labeled standard materials or analogs of the analytes. Considered the best choice to adjust matrix effects, the isotope-labeled ISTD has identical chemical and physical properties to the target analyte. Unfortunately, the isotope-labeled ISTD comes at a high cost, and many compounds do not have certified, isotope-labeled reference materials available on the market.

This study builds on the previous work on adjustment of matrix effects using a universal ISTD, deepoxy-deoxynivalenol (DOM) by Jaderson and Park [10]. Our previous study showed that matrix-induced suppression was substantial; for the majority of the 31 SMs analyzed, we found signal suppression up to greater than 90%. We also showed that DOM as a universal ISTD did not optimally adjust matrix effects for all tested analytes [10].

In the current study using floor dust from schools spiked with standard SMs, we identified the best-performing ISTDs among nine isotope-labeled candidates and unlabeled DOM for each of 36 target analytes, particularly for those with no available isotopes. Next, we tested whether the best-performing analogous ISTDs we had identified could properly adjust for matrix effects in dust samples collected from different schools and could provide more accurate and precise quantification. Proper adjustment of matrix effects will improve our understanding of the associations between exposure and health effects.

## 2. Materials and Methods

### 2.1. Standard Materials and Chemicals

We selected standard materials of 19 microbial SMs and two plant metabolites (linamarin and lotaustralin) that have been previously identified in indoor environments for the study [14]. Since some mycotoxins were reported to be present in indoor building material samples [15], fifteen mycotoxins were also included in the list of 36 total analytes. Table 1 lists the metabolites and mycotoxins evaluated in the study. We were able to obtain nine mycotoxin isotopes labeled with ^13^C that are available on the market. Table 2 lists these nine isotope-labeled mycotoxins and one unlabeled compound that were used as the candidate ISTDs, their suppliers, and abbreviated names. Methanol (>99.9%, LCMS grade), acetonitrile (>99.9%, LCMS grade), acetic acid (≥99.7%, LCMS grade), and ammonium acetate as mobile phase buffer (≥99.0%, LCMS grade) were purchased from Fisher Scientific (Waltham, MA, USA). Ultrapure water was collected through a Millipore Advantage A 10 (EMD Millipore, Burlington, MA, USA) equipped with an LC-pak^®^ polisher with resistivity of 18.2 MΩ cm at 25.0 °C.

### 2.2. Preparation of Standard Solutions for Spiking to Dust

Stock solutions were prepared by mixing standard materials (Table 1) in two different solvents based on their solubility. As 31 of 36 standards were soluble in methanol, standard materials were prepared in methanol at 10 ng/µL concentration as two separate mixed stock solutions. This preparation avoided having too many standards in one solvent, thus making each solution easier to handle. As the remaining five standards (FUB1, NITP, ALT, LIN, and LOT) were best dissolved in acetonitrile/water (1:1 *v*/*v*)-mixed solvent, this was used to prepare the third stock solution of 10 ng/µL concentration. To prepare the final working standard solution for spiking to dust, 400 µL of each of the three stock solutions was diluted to 2 ng/µL with acetonitrile/water (1:1 *v*/*v*). A volume of 125 µL of the working standard solution was then spiked into each dust sample aliquot for the experiments.

### 2.3. Preparation of External Calibration Curves

A series of external standards were prepared from 6 ng/µL for the highest concentration to 5.86 pg/µL for the lowest one by serial dilution in acetonitrile/water (1:1 *v*/*v*). ISTDs including DOM were then added at 30 ng each, except for ^13^C-AFB1 and ^13^C-AFG2, which were added at 3 ng due to much lower concentration in the standard supplied by the manufacturer.

### 2.4. Preparation of Test Dust and Extraction of Metabolites from the Spiked Dust

We selected multiple floor dust samples from two different studies to prepare two sets of samples (i.e., one from each study). The selected dust samples in each set were pooled to create bulk test dust samples—one for the initial and first validation experiment and another for the second validation experiment.

Initial and first validation experiments: The dust samples were collected from 500 elementary school classrooms in a large city in the northeastern region of the United States in the summer of 2015 and were stored at −80.0 °C. Dust was vacuumed from a 2 m^2^ floor area for 5 min using a Li’l Hummer backpack vacuum cleaner (100 CFM, 1.5 horsepower, Pro-Team Inc., Boise, ID, USA) with polyethylene filter socks. Detailed sampling procedures have been previously described [10,16,17]. Of the 500 samples, 10 classroom dust samples with more than a gram of dust from 7 schools were pooled with at least 249 mg of each dust (up to 629 mg) to create a 3.26 g composite sample. This pooling process secured a sufficient amount of dust for the two experiments. The pooled dusts were homogenized using a 360-degree rotary mixer (Appropriate Technical Resources, RKVSD, Laurel, MD, USA) for 2 h. The homogenized dust was portioned into two sets of ten 30-mg samples in 1.5 mL microcentrifuge tubes, one set each for the initial and first validation experiments.

The initial experiment was performed to identify the best-performing ISTDs among the 10 candidates for each of the 36 metabolites. The first validation experiment was performed to verify if recovery rates of spiked metabolites in dust samples using the selected best-performing ISTDs from the initial experiment were reproducible in the repeated experiment using the second set of aliquots from the same pooled dust.

Second validation experiment: This experiment was performed to examine if the selected and verified ISTDs from the initial and first validation experiments, respectively, could properly adjust the matrix effects in different dust (i.e., different sample matrix) collected from different school buildings. Eighty-five initial floor dust samples were collected from two school buildings in a different area in the northeastern region of the United States in May and September 2009. Eight of the 85 dust samples contained more than a gram of dust and thus were selected for the second validation experiment; they were weighed out to at least 133 mg (up to 882 mg) and pooled to make 3150 mg of the second bulk test dust before being homogenized and portioned to ten 30 mg samples in 1.5 mL microcentrifuge tubes.

Sample analysis: One set of five dust sample aliquots (n = 5) for each of the initial and two validation experiments was spiked with 250 ng of each of the 36 external standards, 30 ng of the eight ISTDs (^13^C-CIT, ^13^C-DON, ^13^C-FUB1, ^13^C-NIV, ^13^C-OTA, ^13^C-STEG, ^13^C-ZEA and DOM), and 3 ng of ^13^C-AFB1 and ^13^C-AFG2 ISTDs. A second separate set of five dust aliquots were spiked with only 30 ng of the ISTDs (except for ^13^C-AFB1 and ^13^C-AFG2 spiked at 3 ng) and with no spiked external standards to determine background concentration of the metabolites in pooled dust samples.

Each sample was mixed thoroughly by vortexing for 1 min (Vortex Genie 2, Fisher brand, Pittsburgh, PA, USA) immediately after spiking and then mixed again on a rotary mixer (Appropriate Technical Resources, RKVSD, Laurel, MD, USA) for 30 min before air-drying overnight in a chemical fume hood. For sample extraction, 1 mL of acetonitrile/water/acetic acid (79:20:1, *v*/*v*/*v*) was added to each microcentrifuge tube. The tubes were vortexed for 1 min, sonicated in a water bath (Bransonic M5800H, Branson Ultrasonics Corporation, Danbury, CT, USA) for 15 s, and shaken on a titer plate shaker (Titer Plate Shaker, Lab-Line Instruments, Inc., Melrose Park, IL, USA) for 90 min. After shaking, the extracts were then centrifuged at 3000× *g* for 3 min before aliquoting 900 µL of the supernatant into to a glass centrifuge tube. The extracts were dried under nitrogen (99.999%) using a Turbovap (Zymark, Hopkinton, USA) before reconstituting with 200 µL of mobile phase solvent (methanol/water/acetic acid; 30:69:1, *v*/*v*/*v*). The sample extracts were vortexed for 30 s, capped, and placed on the benchtop for 30 min to dissolve dried deposits before vortexing for another 30 s. Sample extracts were centrifuged again at 3000× *g* for 3 min before transferring 180 µL of the sample extract to high recovery LC vials for LC-MS/MS analysis.

### 2.5. Chromatographic Conditions

We used an Acquity H-Class UPLC (Waters, Milford, MA, USA) installed with a Waters Acquity UPLC BEH C18 column (2.1 mm ID × 150 mm, 1.7 µm particle size, and 130Å pore size) and injected 10 µL of each sample extract twice into the UPLC with chromatographic conditions described by Jaderson and Park [10]. The column and sample tray temperatures were maintained at 50.0 °C and 6.0 °C, respectively, throughout the chromatographic runs. The mobile phase gradient consisted of solvent A (Milli-Q ultrapure water and 1% acetic acid with 10 mM ammonium acetate) and solvent B (methanol and 1% acetic acid with 10 mM ammonium acetate). The mobile phase was set at a flow rate of 200 µL/minute and initialized at 90% solvent A and 10% solvent B from 0 to 30 s. Solvent B was then gradually increased to 50% from 30 s to 1 min, and 97% from 1 to 4.5 min. The 97% solvent B was held for 5 min, and then decreased to 10% from 9.5 to 12 min.

### 2.6. MS Parameters and Transitions

The triple quadrupole mass spectrometer (Waters Xevo TQD, Milford, MA, USA) was operated with an electrospray ionization (ESI) source in the positive-ionization mode for analysis of 31 SMs (Table 3) and in the negative-ionization mode for analysis of 5 SMs (Table 3). The instrument was tuned according to parameters similar to Jaderson and Park [10], at ESI capillary voltage of 1000 V with a source temperature of 150.0 °C and desolvation temperature of 350.0 °C. Laboratory-generated high-purity nitrogen (Peak Scientific Instruments, Bedford, MA, USA) was used with desolvation and cone gas flow rates of 650 and 1.3 L/hr, respectively. The extractor lens was set to 3 V, and the radio frequency lens was set to 2.5 V. The collision cell entrance potential was set to 30 V and exit potential to 30 V. The system was run in multiple reaction monitoring (MRM) mode, with two optimized transitions for each compound (Table 3), except for NITP and ^13^C-CIT where only one reliable transition was identified. Cone voltages were individually optimized for each compound, and collision energy was optimized for each product ion mass transition as in Table 3.

### 2.7. Selection of Best-Performing ISTD for Each Metabolite and Statistical Analysis

Of the 36 metabolites measured in our study (Table 1), only 9 SMs had ^13^C-labeled isotopes available on the market. We used them as isotope-labeled candidate ISTDs and added DOM into the candidate group that was previously used as a universal internal standard [18,19]. As most of the metabolites did not have isotope-labeled ISTDs available, we empirically determined the best-performing ISTD for each metabolite from the initial experiment to adjust matrix effects. To select the best-performing ISTD, we calculated percent recovery rates for each metabolite using each of the ten candidate ISTDs to adjust matrix effects and compared the average recovery rates and coefficient of variation (CV) of ten replicates (some were fewer than ten due to non-detects). Percent CV was calculated by dividing the standard deviation by the mean value of the ten replicates and then multiplying by 100. From this comparison, we selected one ISTD with the average recovery rate closest to 100% and lowest CV as the best-performing ISTD for a particular metabolite.

The percent recovery rate for each sample aliquot was calculated as follows:

(1) $$Recovery\left(\%\right)=\frac{\left(concentration\;in\;spiked\;dust-average\;concentration\;in\;unspiked\;dust\right)}{ex\;pected\;external\;standard\;concentration\;(1250\text{ pg/μL})}\times 100$$

Percent recoveries ranging from 60% to 140% with a CV of <20% are generally considered acceptable in routine analysis [14,20]. Therefore, we categorized 36 metabolites based on performance of the selected ISTD on recovery rates (i.e., capability of adjustment for matrix effects) from the initial experiment into three groups: (1) the metabolites with the most reasonable recoveries (60–140%) and percent CV < 20% were considered ‘acceptable’ with the best-performing ISTDs; (2) the metabolites with reasonable recoveries (60%–140%) but percent CV > 20% were considered ‘marginally acceptable’; and (3) the metabolites outside the reasonable recovery range (<60% or >140%) were considered ‘unacceptable’ [14]. Instrument limit of quantification (LOQ) was measured at 10× the signal-to-noise ratio using the neat standard solution.

## 3. Results

In the unadjusted analysis of spiked samples in the initial experiment, percent recoveries for all the tested metabolites were lower than 60% (unacceptable), except for NEOA (Figure S1 in Supplementary Materials). Percent recoveries for half of them were even less than 20%. The unadjusted percent recoveries ranged from the lowest recovery of 0.1% (18.2% CV) for USN to the highest recovery of 74.9% (3.7% CV) for NEOA.

Table 4 shows the empirically determined, best-performing ISTDs for each metabolite. We expected that 9 of the 36 metabolites that had the available ^13^C-labeled isotopes would match to their own ^13^C-labeled ISTD. However, only four metabolites (CIT, FUB1, OTA, and STEG) matched their own isotopes for the best-performing ISTD. However, the identified best-performing ISTDs for the five mycotoxins (AFB1, AFG2, DON, NIV, and ZEA) were not matched to their own isotopes; their recoveries were poor when their own ^13^C-labeled ISTD was used for their quantification. Thus, other analogous ^13^C-labeled metabolites were selected as the best-performing ISTDs for them (^13^C-labeled OTA for AFB1, AFG2, and DON; ^13^C-labeled CIT for NIV; and ^13^C-labeled STEG for ZEA) (Table 4). Unfortunately, for three metabolites (DON, NIV, and ZEA) of these five, spiking with higher concentrations of 100 ng ^13^C-ISTDs also did not improve poor recoveries. We were not able to spike ^13^C-labeled AFB1 and AFG2 with higher concentrations due to the extremely high cost and the low standard concentration provided by the manufacturer.

**Figure 1.**
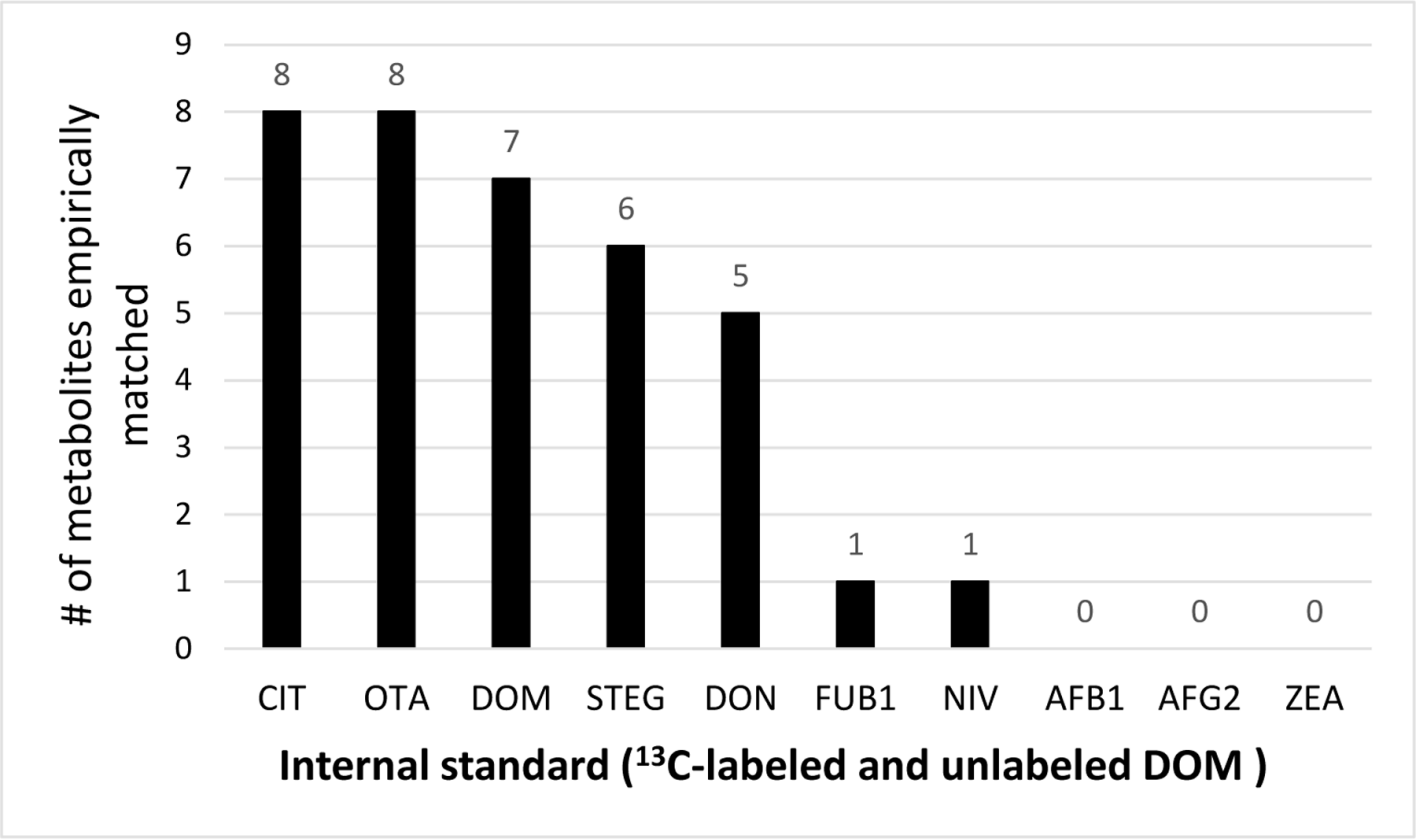
The number of metabolites for which the ^13^C-labeled isotope or DOM was selected as the best-performing ISTD.

^13^C-OTA and ^13^C-CIT were most frequently selected as the best ISTDs for the metabolites, followed by DOM, ^13^C-STEG, and ^13^C-DON (Figure 1). The ISTDs ^13^C-FUB1 and ^13^C-NIV were selected only for one metabolite each (FUB1 and VAL, respectively). Adjustment of matrix effect using the best-performing ISTDs significantly decreased the number of SMs with unacceptable recoveries from 35 SMs (97% of the tested SMs) to 10 SMs (28%) in the initial experiment.

The first validation experiment used the exact same dust sample aliquots under the same analytical conditions as in the initial experiment and produced the same best-performing ISTDs, except for five metabolites: (1) AFG1 for which ^13^C-CIT ISTD was selected

The first validation experiment used the exact same dust sample aliquots under the same analytical conditions as in the initial experiment and produced the same best-performing ISTDs, except for five metabolites: (1) AFG1 for which ^13^C-CIT ISTD was selected from the initial experiment, and ^13^C-OTA from the first and second validation experiments; (2) CYCV for which ^13^C-STEG ISTD was selected from the initial and second validation, and DOM from the first validation; (3) DON for which ^13^C-OTA was selected from the initial and second validation, and DOM from the first validation; (4) INTA for which ^13^C-CIT was selected from the initial and second validation, and ^13^C-NIV from the first validation; and (5) VERO for which ^13^C-CIT was selected from the initial and second validation, and DOM from the first validation (Table 4). The second validation experiment produced the same best-performing ISTDs for 35 metabolites as those from the initial experiment, except for one: AFG1 (^13^C-CIT from the initial experiment and ^13^C-OTA from the second validation).

Determined LOQ ranged from 0.05 pg/µL of extract for ASPG to 62.5 pg/µL of extract for FUB1 and SKY. Table 5 shows the results of the three experiments, average recovery rates, and percent CVs. Twenty-eight of the tested 36 metabolites (78%) fell in either the acceptable or marginally acceptable group from at least one of the three experiments. The initial experiment identified 17 metabolites with the acceptable recoveries, nine with marginally acceptable recoveries, and nine with the unacceptable recoveries with the selected, best-performing ISTDs. One metabolite (SKY) did not give valid results (above the background noise) with any of the ISTDs.

Of the 17 metabolites with acceptable recoveries in the initial experiment, 13 metabolites recorded either acceptable or marginally acceptable recoveries from either one of the validation experiments. The exceptions were FUB1 and NEOA, which were unacceptable from both validations; LIN, which had no valid data from the second validation; and NITP, which had no valid data from either validation. Of the nine metabolites with marginally acceptable recoveries in the initial experiment, eight resulted in either acceptable or marginally acceptable recovery rates in either of the two validations; VAL did not. Of the nine metabolites with unacceptable average recoveries in the initial experiment, two metabolites’ recoveries (DON and ENNB1) were marginally acceptable in either one of the validations, but five were not detected in spiked samples of both validation experiments. SKY was not detected in the spiked samples of the initial experiment but detected with 53–54% recovery in both validation experiments (Table 5).

Generally, the metabolites (NITP, CITRO, LOT, and USN) with higher LOQ values than 15 pg/µL, except for EMOD and AME, had no valid results obtained from both validation experiments. On the other hand, EMOD and AME were among the ones with the lowest recovery rates without adjustment due to the highest matrix effects (Figure S1). Multiple bar plots of adjusted average recovery rates (in Table 5) with error bars are also presented in Figure S2 in the Supplemental Materials.

## 4. Discussion

The current study, as with our previous studies, consistently documents that matrix effects are substantial (signal suppression by more than 80% for many metabolites) in analysis of microbial SMs in indoor floor dust samples with LC-MSMS [6,10]. Vishwanath et al. also reported significant matrix effects that reduced the analytical signals of one third of 186 metabolites spiked in house dust by more than 50% [15]. Therefore, the occurrence of matrix effects in the analysis of microbial SMs in environmental samples using LC-MS/MS seems inevitable [10,20], which indicates that proper adjustment of matrix effects is essential for accurate quantification. Inaccurate measurement of microbial SMs probably produces misclassification in exposures in epidemiological studies, which could potentially confuse the true associations between exposure and health. Our study indicated that the matrix effects for most of the analytes tested may be reasonably compensated by using ^13^C-labeled isotopes or DOM, which were the best-performing ISTDs from our experiments.

Matrix effects are mainly caused by interference in ionization of the target analyte and evaporation of the mobile phase solvent by co-eluted matrix components as they travel from the source capillary to the sample cone inside the electrospray ionization chamber. Inside the chamber, the surface tension of charged liquid droplets in the effluent exiting the high voltage source capillary competes with the electrostatic force on the surface of the droplets, and formation of gas phase ions occurs if the electrostatic force overpowers the surface tension [21,22]. However, some sample chemical components in the droplets, such as surfactants, can decrease the surface tension of charged liquid droplets, which reduces ionization efficiency [21,22]. The evaporation rate of the mobile phase solvent can also be influenced by the heat conductivity of droplets, molecular association between matrix components and target analyte, and vapor pressure of the analyte, which can also be affected by chemical components in the samples [23]. These mechanisms explain why the matrix effects are specific to sample and analyte [24–28]. More details about mechanisms of matrix effects are summarized in Table S1 (supplementary material).

Of the nine isotope ISTDs, ^13^C-labeled CIT and OTA were selected as the best-performing ISTD for 16 metabolites altogether, implying these two isotopes may be reasonable ISTDs for the metabolites with no ^13^C-labeled isotopes available on the market. Both compounds were detected with good sensitivity (LOQs: 3.9 pg/µL for CIT and 0.10 pg/µL for OTA) in our study. We also found that DOM could be considered a reasonable candidate ISTD for some non-mycotoxin SMs.

We observed that five mycotoxins (DON, NIV, ZEA, AFB1, and AFG2) did not match to their own isotope. For DON, NIV, and ZEA, their LOQs were higher (15.6, 7.5, and 25.0 pg/µL, respectively) than those of other metabolites, and signal suppression in their analyses was more than 70%. Therefore, it is possible that a combination of these two factors might have contributed to low detection, which resulted in non-matching to their own isotope as a best-performing ISTD. For AFB1 and AFG2, we had to spike the low concentration of isotopes (tenfold lower than that of the other isotope ISTDs: 0.015 ng/µL versus 0.15 ng/µL) to the dust sample due to the low standard concentration of isotope provided by the manufacturer (1.2 mL of 0.5 µg/mL per vial) at a high cost (about $1000 for one standard vial). The low concentration of isotopes spiked might have contributed to the result of the aflatoxins tested not matching to their own isotope ISTDs. The 0.015 ng/µL ^13^C-aflatoxin ISTDs were detectable in matrix-free samples; however, the isotopes suffered severe peak suppression in matrix-containing sample extracts. Fortunately, four tested aflatoxins had reasonable recoveries (60–134%) when adjusted with ^13^C-OTA. Several studies have shown that aflatoxins had good recovery rates when adjusted for matrix effects with multiple correction methods [29].

Potential reactions of SMs in the mixture may also affect results. For example, FUB1 can react with methanol (solvents used in making the mixtures for spiking and standard curves) to form monomethyl and dimethyl esters [30,31]. This might explain why we had poor recoveries of FUB1 in our experiments. In addition, several metabolites tend to react with other metabolites in a mixture, while others have been reported to have insufficient solubility at high concentrations in a mixture [15,32]. The latter finding implies that the intended concentrations of the affected SMs in a standard mixture may be compromised, which possibly leads to reduced recovery rates. It may be better to separate those SMs into different sets of standard mixtures to avoid such reactions in future studies.

Recovery rate and its CV are important criteria for quality control. Strictly speaking, if the mean recovery rate is in the range of 70–120% with precision (CV) ≤20%, then the data are considered acceptable [33]. However, in routine analysis, recovery rates in the range of 60–140% can be still considered acceptable [14,34,35]. Based on this information, we categorized the SMs into three groups (acceptable, marginally acceptable, and unacceptable) using the criteria of 60–140% recovery rate with 20% CV. Thus, for the analyses of the metabolites in the marginally acceptable group (although their CVs were mostly <50%, Table 5), it would be prudent to have duplicate injections of each sample extract to account for potentially high variance of measurements.

The suggested adjustment method of matrix effects using the best-performing ^13^C-ISTDs found in this study could improve the recovery rate that can be greatly and negatively affected by co-eluted matrix components without proper adjustment as reported by current and previous studies [10,15]. In addition, these matrix effects are analyte- and sample-specific, and yet, there are only limited number of isotopes available on the market. Therefore, finding a perfect ISTD for adjustment of the matrix effects for a specific SM may be very challenging. In such situations, the use of analogous, best-performing ^13^C-ISTDs determined from our study may be beneficial for better quantification of microbial SMs in exposure assessment studies.

We are currently analyzing 150 floor dust samples collected from homes with a history of flooding, and we are using the selected ISTDs from our study to adjust matrix effects, which could be an additional internal validation. It would also be beneficial if the ISTDs determined and the method employed in our study are validated in other studies investigating matrix effects when quantifying secondary metabolites in building dust.

One of the limitations of our study is the high cost of the isotopes; however, this cost may be worthwhile given the importance to most researchers of obtaining high quality scientific data. We tested only 36 SMs, including 15 mycotoxins, and the best-performing ISTDs determined in this study may not be generalized to other SMs not tested.

## 5. Conclusions

Our findings indicated that the use of ^13^C-labeled analogous ISTDs and DOM (empirically determined for the 36 tested metabolites) may be able to properly compensate for matrix effects and to substantially improve accuracy and precision in quantification of many metabolites in school floor dust. ^13^C-labeled CIT and OTA, as well as DOM, were most frequently selected as the best-performing ISTDs for our 36 metabolites. Thus, these ISTDs may be reasonable choices in exposure assessment studies to adjust matrix effects when quantifying some microbial SMs in indoor floor dust. More accurate quantification of microbial SMs, achieved by adjusting for matrix effects in testing, would help us better understand the role of exposure to microbial and non-microbial SMs in the health of occupants in indoor environments.

## Supplementary Material

Supplementary information

## Figures and Tables

**Table 1.** List of the certified reference materials for the tested secondary metabolites

No.	Analyte (SM)	Abbreviation	Supplier*	CAS no.
1	3-Nitropropionic acid	NITP	Sigma-Aldrich	504-88-1
2	Aflatoxin B1	AFB1	Sigma-Aldrich	1162-65-8
3	Aflatoxin B2	AFB2	Fermentek	7220-81-7
4	Aflatoxin G1	AFG1	Sigma-Aldrich	1165-39-5
5	Aflatoxin G2	AFG2	Sigma-Aldrich	7241-98-7
6	Alternariol	ALT	Sigma-Aldrich	641-38-3
7	Alternariol monomethylether	AME	Adipogen	26894-49-5
8	Asperglaucide	ASPG	ChemFaces	56121-42-7
9	Asperphenamate	ASPH	Cayman Chemical	63631-36-7
10	Chaetoglobosin A	CTGA	Adipogen	50335-03-0
11	Citreorosein	CITRO	ChemFaces	481-73-2
12	Citrinin	CIT	Sigma-Aldrich	518-75-2
13	Cyclo(L-Pro-L-Tyr)	CYCT	Bioaustralis	4549-02-4
14	Cyclo(L-Pro-L-Val)	CYCV	Cayman Chemical	2854-40-2
15	Deoxynivalenol	DON	Sigma-Aldrich	51481-10-8
16	Emodin	EMOD	Sigma-Aldrich	518-82-1
17	Enniatin B	ENNB	Cayman Chemical	917-13-5
18	Enniatin B1	ENNB1	Cayman Chemical	19914-20-6
19	Fumonisin B1	FUB1	Sigma-Aldrich	116355-83-0
20	Integracin A	INTA	Santa Cruz	224186-03-2
21	Integracin B	INTB	Santa Cruz	224186-05-4
22	Linamarin	LIN	Cayman Chemical	554-35-8
23	Lotaustralin	LOT	Sigma-Aldrich	534-67-8
24	N-Benzoyl-L-phenylalanine	NBLP	Sigma-Aldrich	2566-22-5
25	Neoechinulin A	NEOA	ChemFaces	51551-29-2
26	Nivalenol	NIV	Fermentek	23282-20-4
27	Ochratoxin A	OTA	Sigma-Aldrich	303-47-9
28	Roquefortine C	ROQC	Santa Cruz	58735-64-1
29	Skyrin	SKY	Sigma-Aldrich	602-06-2
30	Stachybotrylactam	STCH	Santa Cruz	163391-76-2
31	Sterigmatocystin	STEG	Sigma-Aldrich	10048-13-2
32	Usnic Acid	USN	Cayman Chemical	125-46-2
33	Valinomycin	VAL	Sigma-Aldrich	2001-95-8
34	Verrucarin A	VERA	Sigma-Aldrich	3148-09-2
35	Verrucarol	VERO	Sigma-Aldrich	2198-92-7
36	Zearalenone	ZEA	Sigma-Aldrich	17924-92-4

* Sigma-Aldrich, St. Louis, MO, USA; Fermentek, Jerusalem, Israel; Adipogen, San Diego, CA, USA; ChemFaces, Hubei, China; Bioaustralis, Smithfield, NSW, Australia; Cayman, Ann Arbor, MI, USA; Santa Cruz, Dallas, TX, USA.

**Table 2.** List of isotopically labeled ISTD certified reference materials and DOM

No.	Analyte	Abbreviation	Supplier*	CAS no.
1	^13^C-aflatoxin B1	^13^C-AFB1	Romer Labs	1217449-45-0
2	^13^C-aflatoxin G2	^13^C-AFG2	Romer Labs	1217462-49-1
3	^13^C-citrinin	^13^C-CIT	Romer Labs	**518-75-2 (unlabeled)
4	^13^C-deoxynivalenol	^13^C-DON	Romer Labs	911392-36-4
5	^13^C-fumonisin B1	^13^C-FUB1	Romer Labs	1217458-62-2
6	^13^C-nivalenol	^13^C-NIV	Romer Labs	911392-40-0
7	^13^C-ochratoxin A	^13^C-OTA	Romer Labs	911392-42-2
8	^13^C-sterigmatocystin	^13^C-STEG	Romer Labs	**10048-13-2 (unlabeled)
9	^13^C-zearalenone	^13^C-ZEA	Romer Labs	911392-43-3
10	Deepoxy-deoxynivalenol	DOM	Sigma-Aldrich	88054-24-4

* Sigma-Aldrich, St. Louis, MO, USA; Romer Labs, Getzersdorf, Austria

** CAS number of ^13^C-labeled standard is not available

**Table 3.** MS/MS parameters used for 36 metabolites and 10 ISTDs

Metabolite/ISTD	RT (min)	Precursor Ion (m/z)	m/z of product Ion #1	m/z of product Ion #2	Cone Voltage (V)
NITP (neg)	2.05	117.8 [M-H]^−^	45.89 (6)	–*	20
LIN	2.8	265.2 [M+NH_4_]^+^	163.08 (10)	85.03 (20)	34
NIV	3.68	313.22 [M+H]^+^	125.01 (12)	205.06 (12)	25
^13^C-NIV	3.68	328.20 [M+H]^+^	217.08 (12)	186.04 (12)	26
CYCT	4.07	261.09 [M+H]^+^	135.98 (18)	28 (106.99)	34
DON	4.14	297.10 [M+H]^+^	249.10 (10)	203.07 (14)	28
^13^C-DON (pos)	4.14	312.10 [M+H]^+^	216.20 (16)	263.20 (17)	26
^13^C-DON (neg)**	4.14	310.05 [M-H]^-^	261.07 (10)	279.08 (10)	38
CYCV	4.31	197.03 [M+H]^+^	69.98 (22)	169.10 (14)	38
DOM (pos)	4.51	281.16 [M+H]^+^	109.00 (22)	233.11 (10)	26
DOM (neg)**	4.51	339.23 [M-H]^−^	249.05 (10)	279.12 (12)	20
VERO	4.7	267.12 [M+H]^+^	249.15 (8)	231.07 (10)	14
AFG2	4.83	331.04 [M+H]^+^	313.05 (26)	245.05 (30)	50
^13^C-AFG2	4.83	348.10 [M+H]^+^	259.00 (32)	330.00 (36)	56
AFG1	4.96	329.03 [M+H]^+^	243.05 (28)	199.88 (40)	50
AFB2	5.11	315.05 [M+H]^+^	287.05 (28)	259.04 (28)	54
AFB1	5.23	313.10 [M+H]^+^	284.86 (24)	241.11 (40)	62
^13^C-AFB1	5.23	330.10 [M+H]^+^	301.00 (18)	255.10 (26)	54
CIT	5.44	251.05 [M+H]^+^	233.1 (16)	191.00 (26)	28
^13^C-CIT	5.44	264.01 [M+H]^+^	246.05 (15)	–*	34
NBLP	5.49	270.02 [M+H]^+^	104.97 (18)	119.99 (12)	32
NEOA	5.69	324.06 [M+H^]+^	256.05 (10)	268.07 (12)	24
ALT	5.74	258.89 [M+H]^+^	185.00 (30)	127.85 (46)	56
ASPG	5.78	445.17 [M+H]^+^	349.16 (18)	107.03 (38)	40
FUB1	5.78	722.36 [M+H]^+^	352.31 (36)	74.02 (58)	56
^13^C-FUB1	5.78	756.40 [M+H]^+^	374.33 (38)	356.32 (44)	66
CITRO (neg)	6.09	284.95 [M-H]^−^	211.01 (40)	224.07 (32)	66
VERA	6.11	520.38 [M+NH_4_]^+^	249.09 (18)	457.19 (14)	24
ROQC	6.17	390.15 [M+H]^+^	193.00 (26)	322.12 (20)	48
OTA	6.24	404.05 [M+H]^+^	238.96 (22)	(358.06) 14	32
^13^C-OTA (pos)	6.24	424.07 [M+H]^+^	250.03 (26)	109.94 (76)	34
^13^C-OTA (neg)**	6.24	422 [M-H]^−^	174.99 (40)	377.05 (20)	50
ZEA	6.44	321.16 [M+H]^+^	303.13 (14)	189.10 (20)	20
^13^C-ZEA	6.44	337.10 [M+H]^+^	243.15 (22)	185.07 (42)	22
CTGA	6.59	529.16 [M+H]^+^	130.01 (38)	292.05 (24)	26
STEG	6.72	325.03 [M+H]^+^	309.99 (22)	281.05 (34)	56
^13^C-STEG	6.72	343.01 [M+H]^+^	327.06 (28)	297.10 (40)	54
AME (neg)	6.76	270.97 [M-H]^−^	255.99 (22)	182.98 (4)	52
ASPH	6.97	507.19 [M+H]^+^	238.08 (18)	256.09 (12)	34
STCH	7.04	386.19 [M+H]^+^	178.05 (38)	150.16 (50)	66
EMOD (neg)	7.46	268.95 [M-H]^−^	224.99 (24)	240.92 (30)	56
ENNB	7.55	640.44 [M+H]^+^	196.10 (26)	86.05 (70)	60
SKY	7.66	538.99 [M+H]^+^	521.08 (24)	503.87 (40)	64
ENNB1	7.67	654.40 [M+H]^+^	86.05 (62)	196.10 (28)	50
USN (neg)	7.67	343.12 [M-H]^-^	328.00 (22)	259.01 (18)	44
INTB	7.79	587.36 [M+H]^+^	307.15 (18)	166.99 (26)	24
LOT	7.9	262.02 [M+H]^+^	84.94 (22)	162.98 (8)	16
INTA	8.18	629.37 [M+H]^+^	349.18 (14)	289.18 (28)	28
VAL	9.13	1128.65 [M+NH_4_]^+^	172.15 (78)	343.30 (64)	98

See Tables 1 and 2 for the abbreviations of the compound names. m/z = mass-to-charge ratio; CE = Collision Energy; ^13^C = carbon-13 stable-isotope; neg=negative mode; pos=positive mode. The SMs are listed by the order of retention time.

* Only one transition for the MRM was identified.

** Because these three ISTDs were selected for the five SMs with negative mode, they were also analyzed in negative mode.

**Table 4.** Empirically determined best-performing ISTD for each compound.

Compound	Empirically determined ISTD
3-NITP	^13^C-OTA
AFB1*	^13^C-OTA
AFB2	^13^C-OTA
AFG1**	^13^C-OTA
AFG2*	^13^C-OTA
ALT	^13^C-STEG
AME	^13^C-DON
ASPG	DOM
ASPH	DOM
CTGA	^13^C-OTA
CITRO	^13^C-DON
CIT	^13^C-CIT
CYCT	DOM
CYCV**	^13^C-STEG
DON*, **	^13^C-OTA
EMOD	DOM
ENNB	DOM
ENNB1	DOM
FUB1	^13^C-FUB1
INTA**	^13^C-CIT
INTAB	^13^C-DON
LIN	^13^C-CIT
LOT	^13^C-STEG
NBLP	^13^C-CIT
NEOA	^13^C-CIT
NIV*	^13^C-CIT
OTA	^13^C-OTA
ROQC	^13^C-DON
SKY	^13^C-STEG
STCH	^13^C-CIT
STEG	^13^C-STEG
USN	^13^C-DON
VAL	^13^C-NIV
VERA	DOM
VERO**	^13^C-CIT
ZEA*	^13^C-STEG

* AFB1, AFG2, DON, NIV, ZEA: Use of their own ^13^C-labeled metabolite as ISTD did not compensate for matrix effect better than another selected analogous ^13^C-ISTDs.

** Metabolites with inconsistent ISTD selection: AFG1 — The selected ISTD in Table 4 (^13^C-OTA) was the best-performing ISTD for validations 1 and 2, while ^13^C-CIT was the best-performing ISTD for the initial experiment. CYCV, DON, INTA, and VERO — The selected ISTDs in Table 4 were the best-performing ones from the initial and validation 2 experiments, while DOM, DOM, ^13^C-NIV, and DOM were, respectively, selected as the best-performing ISTDs for them from the validation 1 experiment.

**Table 5.** Average percent recovery rates and percent CVs of up to ten measurements (five sample aliquots and duplicate injections per aliquot) for the initial experiment and for the first and second validation experiments by metabolite.

		Initial experiment	First validation experiment		Second validation experiment	
		Dust sample A	Dust sample A		Dust sample B	

Metabolite group	LOQ (pg/µL)	Mean (CV)	Mean (CV)	Difference in recovery (Validation 1 – Initial)	Mean (CV)	Difference in recovery (Validation 2 – Initial)
*1. Seventeen metabolites with acceptable average recoveries from the initial experiment*						
AFB1	0.8	107.2 (9.5)*	97.4 (27.8)**	−9.8	116.9 (27.9)**	9.7
AFB2	1.3	96.3 (9.9)*	107.5 (28.9)**	11.2	108.5 (24.6)**	12.2
AFG1	3.9	87.7 (6.5)*	54.9 (8.2)^†^	−32.8	132.3 (59.9)**	44.6
AFG2	1.1	112.6 (9.8)*	114.5 (29.3)**	1.9	60 (56.3)**	−52.6
ALT	10	133.6 (18.1)*	-^††^	-	63.4 (60.7)**	−70.2
ASPG	0.05	77.5 (20.5)*	88.2 (15.5)*	10.7	91.6 (32)**	14.1
CIT	3.9	116.3 (5.5)*	122 (6.5)*	5.7	106.1 (7.1)*	−10.2
CTGA	2.5	89 (8)*	71.4 (22.8)**	−17.6	53.9 (40.1)^†^	−35.1
CYCT	2	99 (18.6)*	124.7 (19.8)*	25.7	79.2 (22)**	−19.8
FUB1	62.5	101.8 (12.5)*	13.7 (35.4)^†^	−88.1	37.5 (41.7)^†^	−64.3
LIN	7.5	67.5 (17.4)*	50.7 (28.2)^†^	−16.8	-^††^	-
NBLP	0.33	116.9 (8)*	108.7 (12.8)*	−8.2	134.6 (35.8)**	17.7
NEOA	2.6	95.6 (6.3)*	154.4 (8.7)^†^	58.8	161.6 (172.5)^†^	66
NITP	42	94.0 (3.9)*	-^††^	-	-^††^	-
OTA	0.1	111 (9.8)*	112.3 (25.4)**	1.3	164.1 (34.8)^†^	53.1
STCH	1.3	88.9 (6.7)*	90 (5.4)*	1.1	53.1 (49.4)^†^	−35.8
VERO	31	69.1 (17.9)*	40.9 (12.8)^†^	−28.2	108.8 (45.3)**	39.7

*2. Nine metabolites with marginally acceptable average recoveries from the initial experiment*						
CYCV	5.7	120.4 (59.1)**	189.5 (37.3)^†^	69.1	84.8 (25.6)**	−35.6
ENNB	0.9	72.2 (23.4)**	73.5 (12.7)*	1.3	111 (45.9)**	38.8
INTA	1.3	60.2 (31.6)**	85.2 (77.6)**	25	38.5 (47.4)^†^	−21.7
INTB	2.5	64.7 (61.3)**	-^††^	-	64.9 (70.1)**	0.2
NIV	7.5	77.3 (46.7)**	76.4 (46.7)**	−0.9	109.4 (30.4)**	32.1
STEG	0.1	118.5 (20.1)**	135.6 (25.9)**	17.1	47.6 (28.5)^†^	−70.9
VAL	3.5	81.2 (83.2)**	33.8 (85.5)^†^	−47.4	33.2 (51)^†^	−48
VERA	1.3	116.3 (21.3)**	122.2 (13.7)*	5.9	50 (53.9)^†^	−66.3
ZEA	25	83.9 (27)**	58.8 (29.2)^†^	−25.1	65.4 (50.8)**	−18.5

*3. Nine metabolites with unacceptable average recoveries from the initial experiment*						
AME	2.6	26.1 (53.4)^†^	-^††^	-	-^††^	-
ASPH	3.9	2.7 (53.2)^†^	4.2 (80.5)^†^	1.5	15.9 (77.2)^†^	13.2
CITRO	42	33.8 (63.0)^†^	-^††^	-	-^††^	-
DON	16	56.6 (15.6)^†^	64.9 (35.8)**	8.3	108.7 (23.2)**	52.1
ENNB1	1.7	41.4 (26.1)^†^	52.2 (14.4)^†^	10.8	67.5 (50)**	26.1
EMOD	0.7	7.7 (14.0)^†^	-^††^	-	-^††^	-
LOT	25	162.3 (61.6)^†^	-^††^	-	-^††^	-
ROQC	0.16	22 (48.3)^†^	24.5 (75.2)^†^	2.5	35.7 (73)^†^	13.7
USN	16	10.7 (61.3)^†^	-^††^	-	-^††^	-

*4. No valid results obtained from the initial experiment*						
SKY	62.5	-^††^	53.3 (102.3)^†^	-	53.8 (31.4)^†^	-

Superscript value

*= acceptable recoveries

**= marginally acceptable recoveries

†= unacceptable recoveries

†† = no quantifiable peak detected/no data.

## Data Availability

The data presented in this study are available on request from the corresponding author.
