# Supplementary material for: Adjustment of Matrix Effects in Analysis of 36 Secondary Metabolites of Microbial and Plant Origin in Indoor Floor Dust Using Liquid Chromatography-Tandem Mass Spectrometry
Source: Buildings (Basel). Author manuscript; Available in PMC 2024 Apr 22. (PMC11034732; doi:10.3390/buildings13051112)
Supplement: Supplementary information [file NIHMS1973835-supplement-Supplementary_information.pdf]

# *Supplementary Material*

Adjustment of matrix effects in analysis of 36 secondary metabolites of microbial and plant origin in indoor floor dust using liquid chromatography-tandem mass spectrometry

Cornelius Rimayi and Ju-Hyeong Park*

Respiratory Health Division, National Institute for Occupational Safety and Health, Centers for Disease Control and Prevention, Morgantown, WV 26505, United States

* Corresponding author: Ju-Hyeong Park

Tel.: 304-285-5967; email: gzp8@cdc.gov

Key words: Indoor Floor dust; Matrix effect adjustment; Microbial secondary metabolite (MSM); Recovery; Validation

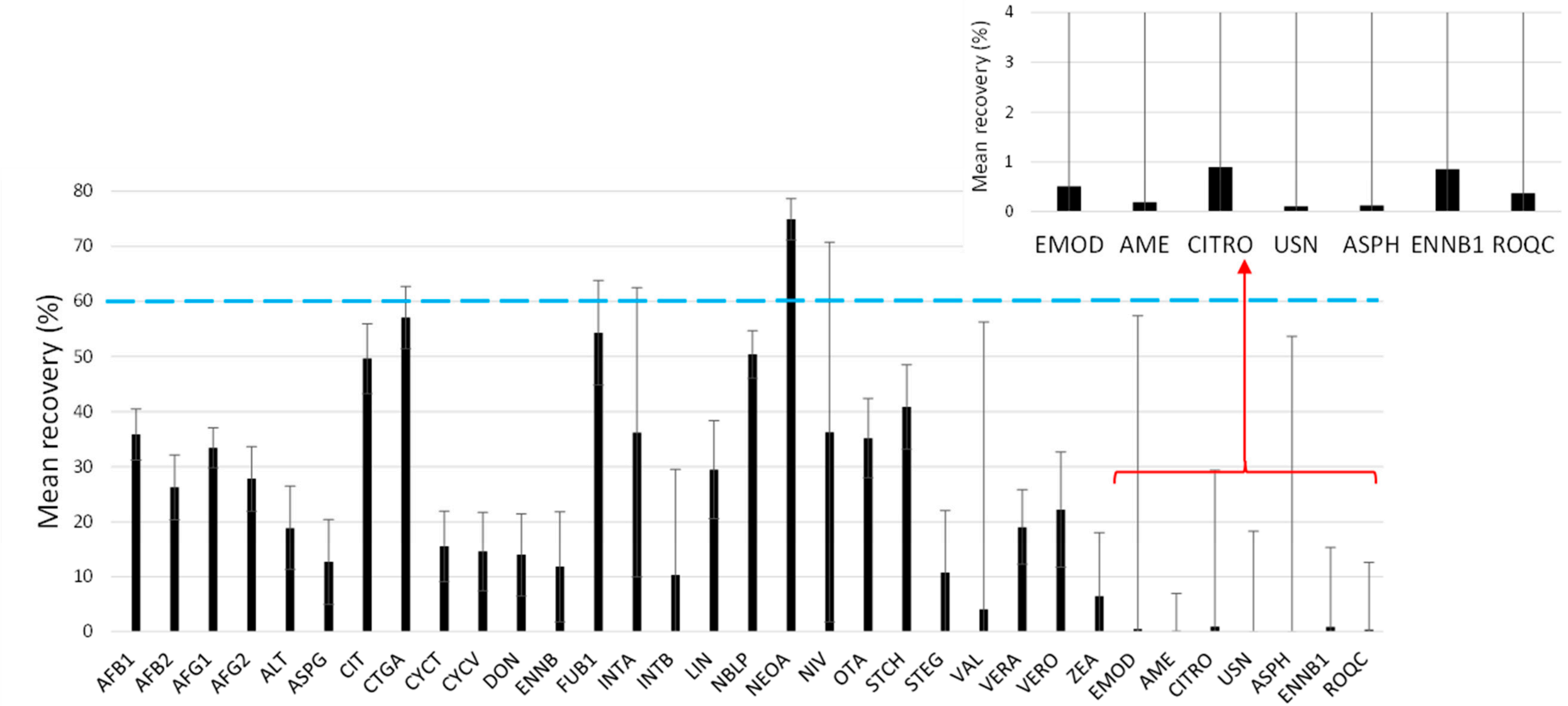


Figure S1. Mean percent recovery rates and standard deviations of SMs (insert: < 1% mean percent recoveries) when matrix effects were unadjusted in the initial experiment. Blue line shows acceptable recovery threshold (≥ 60%).

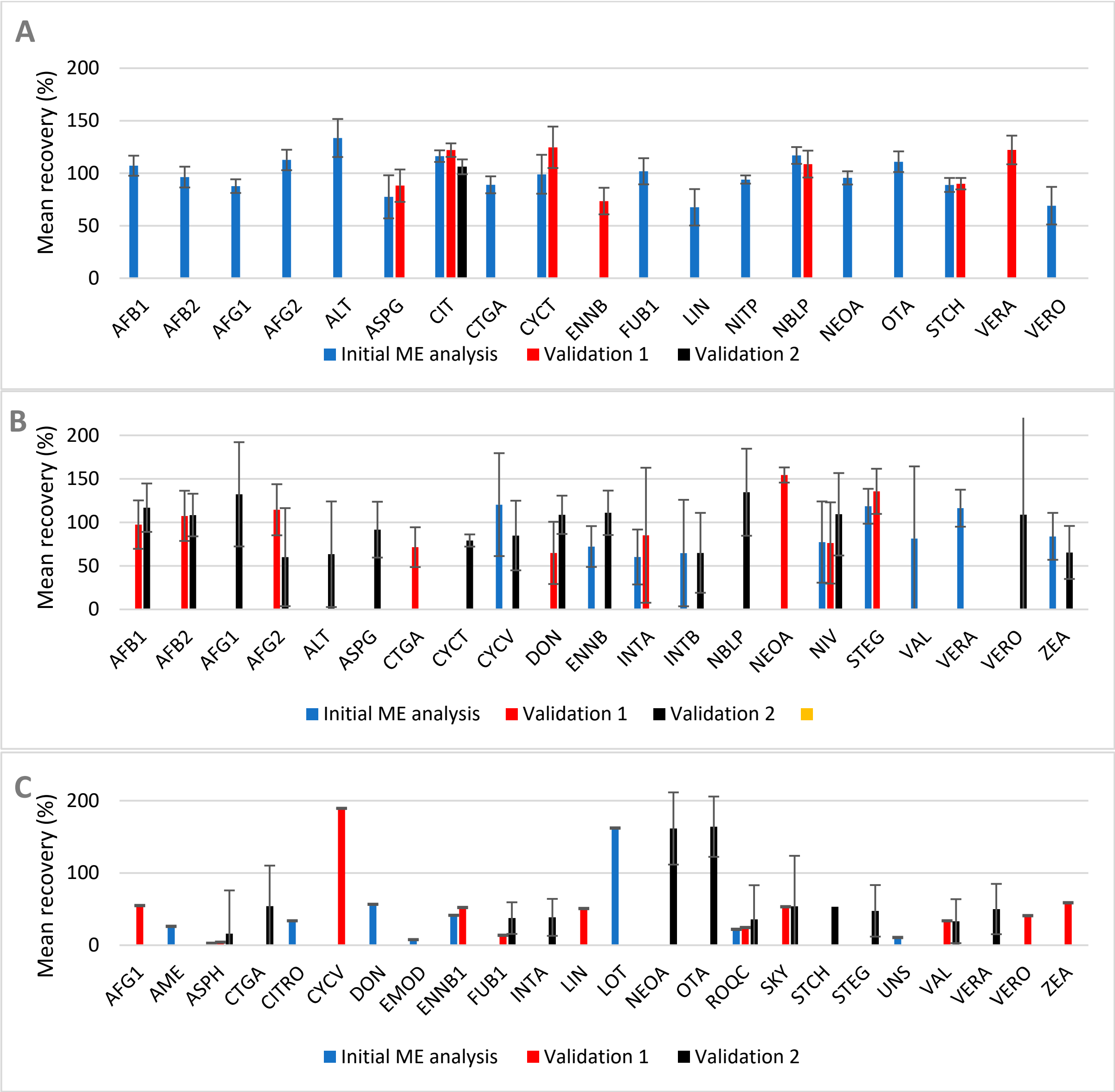


Figure S2. Mean percent recovery rates and standard deviations of SMs from the three experiments when matrix effects were adjusted with the best performing ISTDs. A: metabolites for which recoveries were in 60–140% with <20% CV; B: recoveries in 60–140% but % CV > 20; and C: recoveries <60 or > 140%. ME = matrix effects

Table S1. Proposed mechanisms, results and scenarios of matrix effects

| Matrix effect occurrence site | Mechanism of Matrix Effect | Result of Matrix Effect |
|---|---|---|
| Mass spectrometer | Coeluting sample matrix components with same transitions masking or enhancing signals of target analyte [1, 2] | Matrix-induced enhanced or suppressed signal; difficult integration conditions such as peak shape distortion; elevated background; poor and inconsistent signal reproducibility between replicate injections [2]; wider peaks on tailing or fronting sides of background or matrix peaks |
| High voltage capillary | Viscous materials co-eluted with sample matrix components adversely hinder effective nebulization of mobile phase by a combination of pneumatic force (from high velocity nitrogen gas) and electrospray voltage (electric field) | Diminished efficiency in both nebulization and Coulomb explosion of liquid droplet, depending on matrix component concentration |
| High voltage capillary and liquid droplet | Both electrolyte salts and non-conducting inorganic matrix components such as carbonates/sulfates in high concentrations alter both the drying rate and electrical conductivity of the mobile phase exiting the high voltage capillary as well as liquid droplets [2, 3]. For matrix based extracts, this will ultimately have a significant effect on the dispersion of solute free ions in the gas phase, influencing the chemical composition and abundance of desolvated analyte ions in the gas phase [4] | Inefficient drying and subsequent low ionization of target analytes due to a combination of the shift in effective drying gas flow, drying temperature and the low conductivity of liquid droplets |
| Liquid droplet | Coeluting sample matrix components compete for limited charge space on the surface of the nebulized liquid droplet [2, 5] | Diminished charge capture by analytes of interest due to more charging capture by matrix components. Lower proportion of target ions ejection from surface of charged liquid droplet [6] |
| Liquid droplet | Coeluting matrix components may alter the surface tension holding the liquid droplet together, thereby altering the degree of Coulomb force (force of | Formation of gas phase ions in matrix extracts occurs at different rates and efficiency as with matrix-free analytical standard: (i) in case of lower surface tension, surfactant-like matrix |

| | | |
|---|---|---|
| | repulsion between the like charges) required to overcome the surface tension (Rayleigh limit) [7]. | components rapidly collapse the liquid droplets as they can no longer easily contain the Coulombic force [7, 8]; and (ii) in case of higher surface tension resulting from matrix components, larger and less frequent Coulombic explosions are generated, which disperses the sample across a much larger area beyond the MS sampling cone, causing analyte loss [7, 8]. |
| Liquid droplet | Partitioning of target analytes strongly onto non-volatile matrix components. Non-volatile components may reduce the electrical conductivity of the nebulized liquid droplets, resulting in reduction in the ability of the charged droplets to generate enough electrostatic stress (Coulomb force [7]) on the surface on the liquid droplet required to generate a coulombic explosion (burst of the liquid droplet) as well as generate coulombic fission events to generate smaller liquid droplets. | Inefficient ionization of target analytes due to low coulombic explosions and coulombic fission. |
| Liquid droplet and gas phase | Before undergoing Coulomb fission (according to Fenn's model of ion formation), large nebulized liquid droplets contain several different analytes of different shapes and sizes [7]. As a result of matrix components in the large liquid droplet , there is more competition for proton/adduct transfer between the analyte of interest and the coeluting analytes. This competition may be carried over to the gas phase. | Diminished efficiency of proton/adduct acceptance/release by the analyte of interest |

References


1. Tisler, S., D. I. Pattison and J. H. Christensen. "Correction of matrix effects for reliable non-target screening lc–esi–ms analysis of wastewater." *Analytical Chemistry* 93 (2021): 8432-41. 10.1021/acs.analchem.1c00357. https://doi.org/10.1021/acs.analchem.1c00357.
2. Nasiri, A., R. Jahani, S. Mokhtari, H. Yazdanpanah, B. Daraei, M. Faizi and F. Kobarfard. "Overview, consequences, and strategies for overcoming matrix effects in lc-ms analysis; a critical review." *Analyst* (2021):
3. Page, J. S., R. T. Kelly, K. Tang and R. D. Smith. "Ionization and transmission efficiency in an electrospray ionization–mass spectrometry interface." *Journal of the American Society for Mass Spectrometry* 18 (2007): 1582-90. https://doi.org/10.1016/j.jasms.2007.05.018. https://www.sciencedirect.com/science/article/pii/S1044030507004357.
4. Nguyen, S. and J. B. Fenn. "Gas-phase ions of solute species from charged droplets of solutions." *Proc Natl Acad Sci U S A* 104 (2007): 1111-7. 10.1073/pnas.0609969104.
5. Skillman, B. and S. Kerrigan. "Identification of suvorexant in blood using lc–ms-ms: Important considerations for matrix effects and quantitative interferences in targeted assays." *Journal of Analytical Toxicology* 44 (2019): 245-55. 10.1093/jat/bkz083. https://doi.org/10.1093/jat/bkz083.
6. Fenn, J. B. "Ion formation from charged droplets: Roles of geometry, energy, and time." *Journal of the American Society for Mass Spectrometry* 4 (1993): 524-35. 10.1016/1044-0305(93)85014-O. https://doi.org/10.1016/1044-0305(93)85014-O.
7. Banerjee, S. and S. Mazumdar. "Electrospray ionization mass spectrometry: A technique to access the information beyond the molecular weight of the analyte." *International Journal of Analytical Chemistry* 2012 (2012): 282574. 10.1155/2012/282574. https://doi.org/10.1155/2012/282574.
8. Kirjavainen, P. V., M. Täubel, A. M. Karvonen, M. Sulyok, P. Tiittanen, R. Krska, A. Hyvärinen and J. Pekkanen. "Microbial secondary metabolites in homes in association with moisture damage and asthma." *Indoor Air* 26 (2016): 448-56. 10.1111/ina.12213.
